# SBF-1 inhibits contact hypersensitivity in mice through down-regulation of T-cell-mediated responses

**DOI:** 10.1186/s40360-019-0377-8

**Published:** 2019-12-21

**Authors:** Wei Chen, Xianying Fang, Yuan Gao, Ke Shi, Lijun Sun, Biao Yu, Qiong Luo, Qiang Xu

**Affiliations:** 1 State Key Laboratory of Pharmaceutical Biotechnology, School of Life Sciences, Nanjing University, 163 Xianlin Avenue, Nanjing, 210023 China; 2 College of Chemical Engineering, Nanjing Forestry University, Nanjing, 210037 China; 3 State Key Laboratory of Bioorganic and Natural Products Chemistry, Shanghai Institute of Organic Academy, Shanghai, 200032 China

**Keywords:** SBF-1, Activated T lymphocytes, Immunosuppression, Contact hypersensitivity

## Abstract

**Background:**

T lymphocytes play an important role in contact hypersensitivity. This study aims to explore the immunosuppressive activity of SBF-1, an analog of saponin OSW-1, against T lymphocytes in vitro and in vivo.

**Methods:**

Proliferation of T lymphocytes from lymph nodes of mice was determined by MTT assay. Flow cytometry analysis was performed to assess T cell activation and apoptosis. Levels of cytokines were determined by PCR and ELISA. BALB/c mice were sensitized and challenged with picryl chloride and thickness of left and right ears were measured.

**Results:**

SBF-1 effectively inhibited T lymphocytes proliferation induced by concanavalin A (Con A) or anti-CD3 plus anti-CD28 at a very low dose (10 nM) but exhibited little toxicity in non-activated T lymphocytes at concentrations up to 10 μM. In addition, SBF-1 inhibited the expression of CD25 and CD69, as well as he phosphorylation of AKT in Con A-activated T cells. SBF-1 also induced apoptosis of activated T cells. In addition, SBF-1 also downregulated the induction of the T cell cytokines, IL-2 and IFN-γ in a dose-dependent manner. Furthermore, SBF-1 significantly suppressed ear swelling and inflammation in a mouse model of picryl chloride-induced contact hypersensitivity.

**Conclusions:**

Our findings suggest that SBF-1 has an unique immunosuppressive activity both in vitro and in vivo mainly through inhibiting T cell proliferation and activation. Its mechanism appears to be related to the blockage of AKT signaling pathway.

## Background

T cells are a key component of the adaptive immune system and they play an important role in protecting the host from invading pathogens [1]. Naïve T cells are activated by the interaction between T cell receptor (TCR) and antigenic peptides presented by major histocompatibility complex (MHC) on the antigen presenting cells, and then trigger protective immune responses [2, 3]. However, when activated inappropriately, T cells contribute to a wide spectrum of disease [4, 5]. Activated T cells can be phenotypically recognized by the up-regulation of cell surface markers using monoclonal antibodies and flow cytometry [6]. CD69 is a co-stimulatory molecule and is rapidly detectable within a few hours after ligation of the TCR/CD3 complex [7]. CD25 is also expressed on activated T cells, but its appearance on membrane is slower than that of CD69 [6].

Contact hypersensitivity (CHS) is induced by topical skin immunization with pathogens, allergens, or toxic chemical stimulations [8] and it is recognized as a classic T cell-mediated immune response [9]. CHS consists of two distinct phases, an induction phase and an elicitation phase. The induction phase is primed by the application of a hapten, and the elicitation phase is initiated after re-exposure to the same hapten [10, 11]. The activation and proliferation of T lymphocytes play crucial roles in both stages.

Clinically, immunosuppressive agents such as dexamethasone and cyclosporine A are used to counteract CHS. However, because of their nonselective inhibition on non-activated T lymphocytes and activated T lymphocytes, most of these agents are reported to cause severe side effects including immunosuppression and the increased risk of infection [12, 13]. Thus, there is an urgent need for effective immunosuppressive agents with high selectivity and low toxicity.

SBF-1 is one of the 23-oxa-analogs of saponin OSW-1, which inhibits the growth of human cancer cell lines more effectively than OSW-1 [14]. Usually, anti-cancer drugs are known to cause severe adverse effects such as immune system damage. However, little is known about SBF-1’s effect on immune system. Here, we found that SBF-1, exhibited a strong ability to inhibit activated T lymphocytes function in vitro and in vivo, which is possibly associated with its suppression of AKT phosphorylation.

## Methods

### Mice

Specific pathogen-free, 6–8 weeks old female BALB/c mice were obtained from Model Animal Genetics Research Center of Nanjing University (Nanjing, China). They were maintained with free access to pellet food and water in plastic cages 21 ± 2 °C and kept on a 12 h light/dark cycle. Animal welfare and experimental procedures were carried out strictly in accordance with the Guide for the Care and Use of Laboratory Animals (National Institutes of Health, the United States). All efforts were made to minimize animals’ suffering and to reduce the number of animals used. The animal protocols in this study were approved by the medical ethical committee of Nanjing University.

### Reagents

SBF-1 was synthesized by Prof. Biao Yu, a coauthor, as previously reported [14]. SBF-1 was dissolved in DMSO and stored at − 20 °C. For in vitro studies, the stock solution concentration was 20 mM. For in vivo studies, the stock solution concentration was 10 mg/ml. The final DMSO concentration did not exceed 0.1% throughout the study. Concanavalin A (Con A) was purchased from Sigma-Aldrich (St. Louis, MO). Purified anti-mouse CD3 and anti-mouse CD28were purchased from BD Biosciences (San Jose, CA). 3-(4,5-Dimethylthiazol-2-yl)-2,5-diphenyltetrazolium bromide (MTT) and Annexin V/PI kit were purchased from Sunshine Biotechnology (Nanjing, China). Fetal bovine serum (FBS), RPMI 1640, penicillin and streptomycin were purchased from Life Technology (Carlsbad, CA). FITC anti-mouse CD25 antibody and FITC anti-mouse CD69 antibody were purchased from eBioscience (San Diego, CA). IFN-γ ELISA kit and IL-2 ELISA kit were purchased from Dakewe Biotech (Beijing, China). Primary antibodies against AKT (pS473) and AKT were purchased from Cell Signal Technology (Beverly, MA). Antibody against GAPDH was purchased from Santa Cruz Biotechnology (Santa Cruz, CA). Picryl chloride (PCl) was purchased from Wako Pure Chemical Industries (Osaka, Japan).

### T lymphocytes culture and proliferation assay

Mouse T lymphocytes were obtained from lymph nodes of BALB/c mice. The Pan T cell Isolation Kit (Miltenyi Biotec, Bergisch Gladbach, Germany) was used to obtain purified T cells. Flow cytometric analysis showed that the purity was over 95%. T cells were then incubated in RPMI 1640 supplemented with 100 U/ml penicillin, 100 μg/ml streptomycin and 10% FBS in a humidified 5% (v/v) CO_2_ atmosphere at 37 °C. Purified T lymphocytes were cultured in 96-well plates (3 × 10^5^ cells/well) and stimulated with Con A or anti-CD3/anti-CD28 for 72 h. Cell growth was then assessed by MTT assay or [^3^H]-thymidine uptake assay.

### MTT assay

Cell viability was assessed by MTT assay. MTT (4 mg/ml in PBS, 20 μl per well) was added to each well. After 4 h of additional incubation, the culture media was removed and 200 μl DMSO was added to dissolve the crystals. The absorption values at 570 nm were determined using a microplate reader.

### [^3^H]-thymidine uptake assay

Cell proliferation was assayed by incorporation of [methyl-^3^H] thymidine (ICN Pharmaceuticals, Costa Mesa, CA) at 0.5 μCi/well during the last 8 h of incubation, and the uptake was measured as counts per minute (c.p.m.) by a liquid scintillation counter.

### Flow cytometry analysis

T cells (5 × 10^5^ cells/well) were isolated from lymph node of BALB/c mice and seeded into 6-well plates. The cells were then incubated with SBF-1 and stimulated simultaneously with Con A (5 μg/ml). Twenty four hours later, the harvested cells were washed twice with PBS and then stained with FITC-anti-mouse CD25 or FITC-anti-mouse CD69 for 30 min at 4 °C in the dark. Finally, cells were washed with PBS to remove excess stains and analyzed by FACS Calibur flow cytometer (Becton Dickinson, San Jose, CA) using CellQuest software. Apoptosis was determined by Annexin V/PI staining and analyzed by FACS Calibur flow cytometer.

### Semi-quantitative PCR

Total RNA was extracted from cells using Trizol Reagent (Invitrogen, Carlsbad, CA). One microgram of RNA was reversely transcribed to cDNA. PCR primer sequences were as follows: GAPDH, forward 5′-AACGACCCCTTCATTGAC and reverse 5′- CCACGACATACTCAGCAC; IFN-γ, forward 5′- TGAACGCTACACACTGCATC and reverse 5′- CCATCCTTTTGCCAGTTCCTC; IL-2, forward 5′-CTACAGCGGAAGCACAGC and reverse 5′-TCCTCAGAAAGTCCACCA. PCR was performed at 94 °C for 30 s, 55 °C for 45 s, and 72 °C for 45 s. The cycling number was 28 for GAPDH. Others were 30 cycles. PCR products were electrophoresed in 1.5% agarose gel and visualized by means of ethidium bromide staining.

### ELISA assay

IFN-γ and IL-2 levels in cell culture supernatant were measured using ELISA kits according to the manufacturer’s instructions.

### Western blot

Whole cell lysates was prepared using cell lysis buffer (Beyotime, Shanghai, China) containing protease and phosphatase inhibitors. The proteins were then separated by SDS-PAGE and electrophoretically transferred to a PVDF membrane. After blocking with 5% milk for 1 h, the membrane was probed overnight at 4 °C with primary antibodies. On the next day, the membrane was incubated with HRP-conjugated secondary antibodies for 2 h. Detection was performed using the LumiGLO chemiluminescent substrate system (Cell Signaling Technology, Massachusetts, USA).

### Picryl chloride (PCl)-induced contact hypersensitivity

On day 0, mice were distributed into four groups (*n* = 6) according to body weight. And then they were shaved and sensitized by applying 0.1 ml of absolute ethanol containing 1% PCl to the shaved skin on their abdomens. On day 5, these mice were challenged by applying 30 μl of 1% PCl in olive oil to the right ear. After 18 h, ear thickness of left and right ears of all animals were measured using a digimatic micrometer (0.001 mm, Mitutoyo Co., Tokyo, Japan). Ear swelling of each mouse was calculated as follows: right ear thickness – left ear thickness. 5 μg/kg and 10 μg/kg SBF-1 and 10 mg/kg CsA were administered intraperitoneally once daily from day 0 to day 6. Control mice were given vehicle (0.1% DMSO in PBS) instead of SBF-1. After the end of experiment, animals were sacrificed by cervical dislocation and the ear samples were collected.

### Histological analysis

Formalin-fixed, paraffin-embedded ear tissue was cut to a thickness of 5 mm, and the sections were stained with hematoxylin and eosin. Following parameters were assessed: 1: the level of leucocyte infiltration and vascular congestion; 2: the erosion and anabrosis of epidermal cells; 3: inflammation to the ventral side of the ear. The histological scores were assessed from 1 to 4. Final data are the average scores of each animal in the same group, and the higher score means more serious inflammation.

### Statistical analysis

Data are expressed as means ± SEM. Analysis of experiments was performed using one-way ANOVA followed by post-hoc (Tukey’s correction for multiple comparisons).

## Results

### SBF-1 inhibited the proliferation of T cells in a concentration-dependent manner

**Fig. 1:**
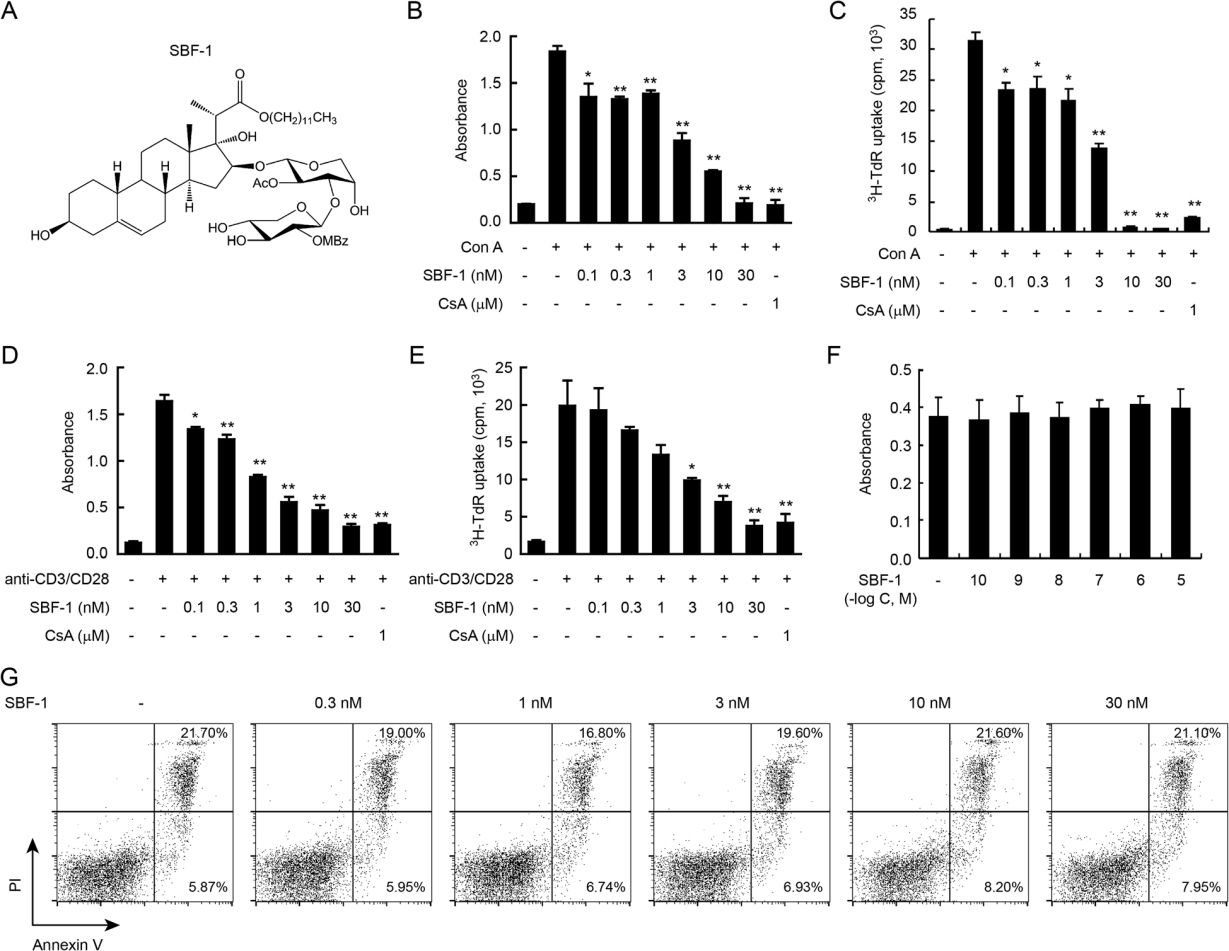
SBF-1 markedly inhibited the proliferation of Con A-activated T cells. **a** The chemical structure of SBF-1. **b**–**e** T cells (3 × 10^5^) were isolated from lymph node of BALB/c mice and then incubated with 0.1–30 nM SBF-1 in the presence of 5 μg/ml Con A **b**–**c** or 1 μg/ml anti-CD3/CD28 **d**–**e** for 72 h at 37 °C. Cell proliferation was measured at 540 nm by MTT uptake assay **b**, **d** or [^3^H]-thymidine uptake assay (**c**, **e**). Data are mean ± SEM of three independent experiments. *P* values were determined by one-way ANOVA with Tukey’s correction. *P < 0.05, **P < 0.01. **f**–**g** T cells (5 × 10^5^) were isolated from lymph node of BALB/c mice and then incubated with SBF-1 for 24 h at 37 °C. Cell viability was measured at 540 nm by MTT uptake assay **f**. Cell apoptosis was analyzed by Annexin V/PI assay staining **g**. Data are mean ± SEM of three independent experiments

The structure of SBF-1 was shown in Fig. 1a. First we performed MTT assay, which is an effective method for testing mitochondrial impairment and correlates quite well with cell proliferation [15]. As shown in Fig. 1b, SBF-1 significantly reduced mitochondrial metabolic activity of Con A-activated T lymphocyte. In addition, in the [^3^H]-thymidine uptake assay, SBF-1 significantly inhibited Con A-induced T lymphocyte proliferation in a concentration-dependent manner (Fig. 1c). Furthermore, because Con A was a non-specific stimulus, we used a more physiological in vitro model with anti-CD3 plus anti-CD28. As shown in Fig. 1d and e, SBF-1 also dose-dependently inhibited T cell proliferation induced by anti-CD3 plus anti-CD28 by MTT assay and [^3^H]-thymidine uptake assay. Notably, SBF-1 at only 10 nM showed excellent anti-proliferation properties, which was 100-fold more potent than cyclosporine A. It was important to notice that SBF-1, at doses from 0.1 to 30 nM, did not affect the survival of non-activated T cells in MTT assay and Annexin V/PI assay (Fig. 1f and g). Cyclosporine A (CsA), which is widely used as an immunosuppressant in the clinical setting, was used as a positive control here. Previous study showed that CsA greatly decreased cell viability both in activated and non-activated T cells [16]. These results suggest that, in contrast to CsA, the immunosuppressive activity of SBF-1 is not caused by cytotoxicity of immune cells.

### SBF-1 suppressed the activation of T lymphocytes and inhibited AKT signaling

**Fig. 2:**
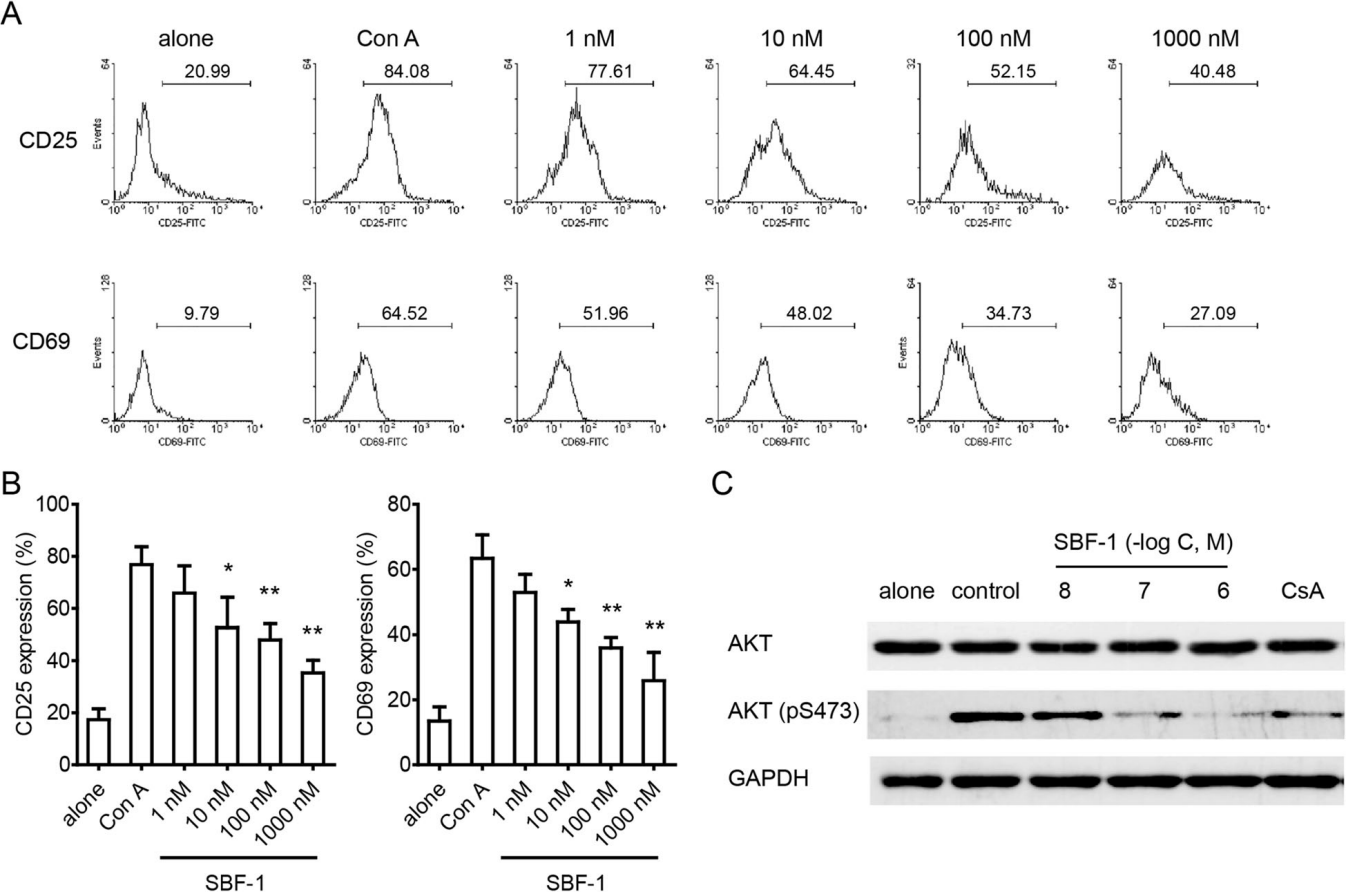
SBF-1 suppressed T cell activation through inhibiting AKT signaling. T cells were isolated from lymph node of BALB/c mice and then incubated with SBF-1 in the presence of 5 μg/ml Con A for 24 h at 37 °C. **a**–**b** Cells were collected and analyzed for CD69 and CD25 expressions by flow cytometry. Data **a** shown here are one of three different experiments with similar results. Data **b** are mean ± SEM of three independent experiments. *P* values were determined by one-way ANOVA with Tukey’s correction. *P < 0.05, **P < 0.01. **c** Cells were harvested and the whole cell extracts were analyzed by western blotting for AKT and the phosphorylation of AKT. Data shown here are one of three different experiments with similar results

CD25 and CD69 are markers of activated T lymphocytes [17]. CD69 can be induced by Con A and rapidly detected on the surface of T cells [18]. CD25 is also expressed on activated T cells, but its appearance on membrane is slower than CD69 [6]. Flow cytometric analysis showed that SBF-1 markedly reduced the percentages of CD25^+^ and CD69^+^ T cells which were up-regulated after incubation with Con A for 24 h (Fig. 2a). To further explore the potential molecular mechanism of SBF-1, western blotting assay was performed. It is known that the AKT signaling pathway is a central regulator in mammalian cells which controls cell growth and proliferation [19]. As shown in Fig. 2b, the phosphorylation level of AKT was suppressed by SBF-1 in a dose-dependent manner in Con A-activated T lymphocytes.

### SBF-1 induced apoptosis and reduced T cell cytokines production in activated T cells

**Fig. 3:**
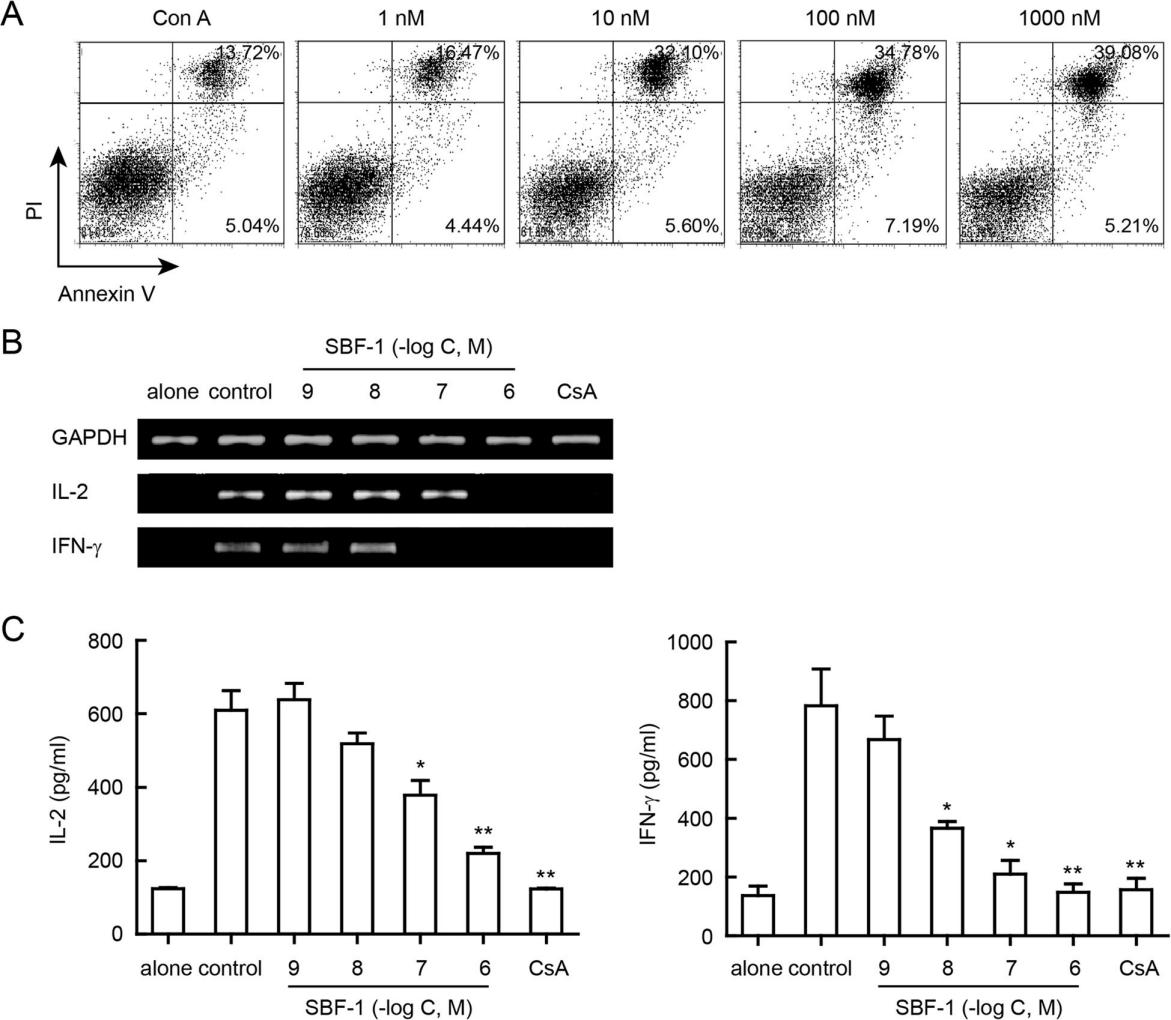
SBF-1 induced apoptosis and inhibited T cell cytokines production of activated T lymphocytes. **a** T cells were isolated from lymph node of BALB/c mice and then activated with 5 μg/ml Con A for 24 h at 37 °C. After being incubated with SBF-1 for 24 h, cells were collected and stained with Annexin V/PI to analyze apoptosis by flow cytometry. Data shown here are one of three different experiments with similar results. **b**–**c** T cells were isolated from lymph node of BALB/c mice and then incubated with SBF-1 in the presence of 5 μg/ml Con A for indicated time at 37 °C. **b** T cells were incubated for 24 h, and then they were collected. The mRNA expression of IL-2 and IFN-γ were determined by RT-PCR. Data shown here are one of three different experiments with similar results. **c** T cells were incubated for 72 h, and then cell culture supernatants were harvested. The levels of IL-2 and IFN-γ were measured by ELISA assay. Data are mean ± SEM of three independent experiments. *P* values were determined by one-way ANOVA with Tukey’s correction. *P < 0.05, **P < 0.01

Next, we analyzed the effect of SBF-1 on apoptosis of activated T lymphocytes. Previously we have found that SBF-1 did not affect survival of non-activated T cells in MTT and Annexin V/PI assay (Fig. 1f and g). However, as shown in Fig. 3a, treatment with SBF-1 significantly induced apoptosis of Con A-activated T cells by Annexin V/PI assay, indicating that SBF-1 only induced apoptosis of activated T lymphocytes, not non-activated T lymphocytes. In addition, the levels of T cell cytokines, such as IL-2 and IFN-γ, were measured by PCR and ELISA assays. Analysis of mRNA and culture supernatant of Con A-activated T cells showed that the production and secretion of IL-2 and IFN-γ were considerably increased after stimulation. As shown in Fig. 3b and c, SBF-1 concentration dependently inhibited these two cytokines levels in both mRNA and protein levels.

### SBF-1 ameliorated ear swelling in mice with contact hypersensitivity induced by picryl chloride

**Fig. 4:**
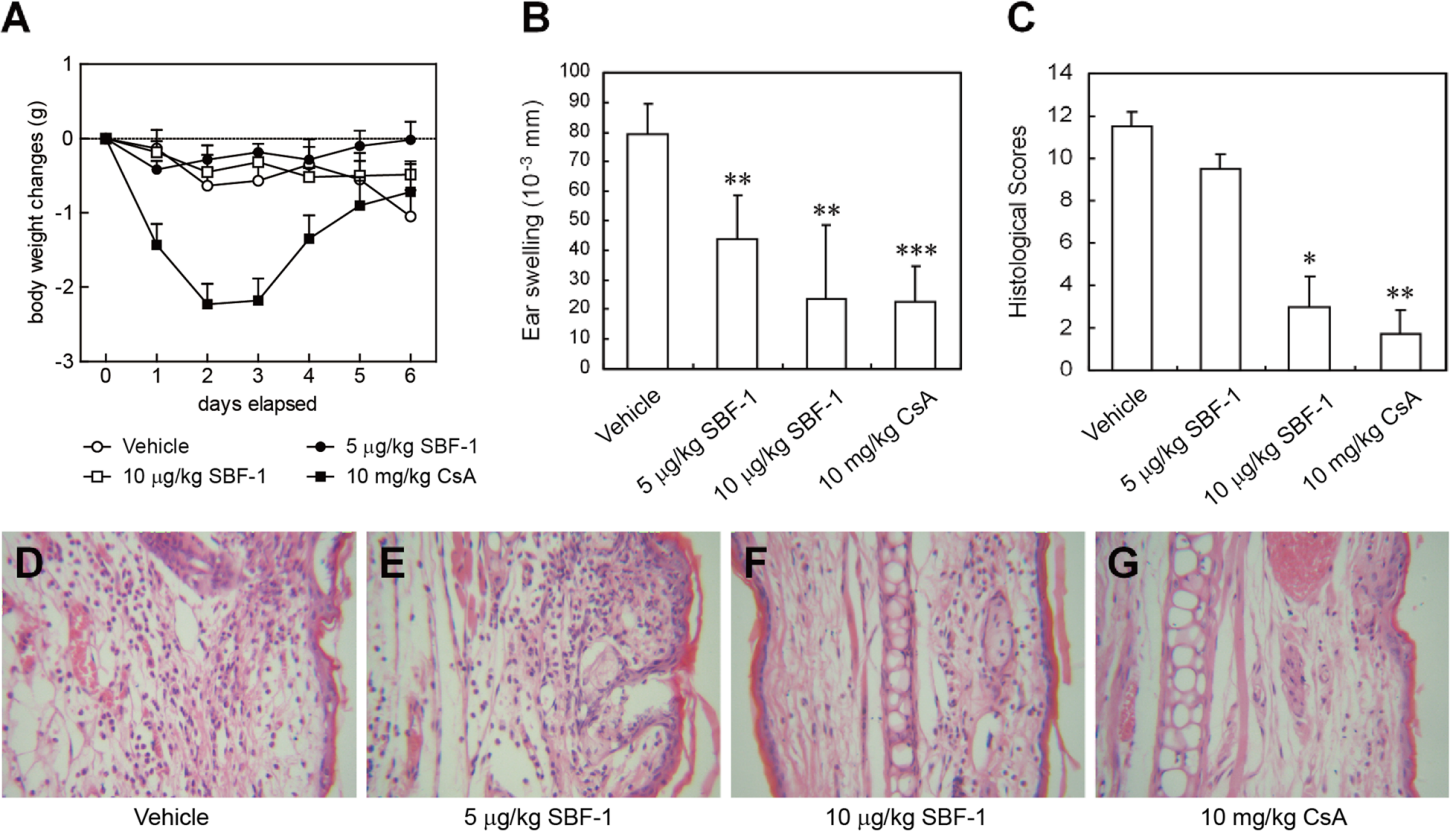
SBF-1 ameliorated ear swelling in mice with contact hypersensitivity induced by picryl chloride. Contact hypersensitivity was induced in BALB/c mice by PCl. SBF-1 or vehicle control was administered i.p. daily from day 0. **a** Body weight changes. **b** Ear swelling. **c**–**g** Ear tissues harvested from vehicle- or SBF-1-treated mice on day 6 were analyzed for degree of inflammation by H&E (original magnification × 200). **c** The pathological score. Data are mean ± SEM of six animals per group. *P* values were determined by one-way ANOVA with Tukey’s correction. *P < 0.05, **P < 0.01. **d**–**g** Representative photographs of each group: Vehicle **d** 5 μg/kg SBF-1 **e** 10 μg/kg SBF-1 **f** 10 mg/kg cyclosporine A **g**

In order to assess the immunosuppressive property of SBF-1 in vivo, contact dermatitis was induced in BALB/c mice with picryl chloride (also referred to as trinitrochlorobenzene, TNCB). The mice were sensitized with PCl on day 0 and challenged with PCl on day 5. SBF-1 was intraperitoneally injected once daily until scheduled euthanasia on day 6. As shown in Fig. 4b, the ear swelling was dose-dependently inhibited by SBF-1 at 5 and 10 μg/kg without obvious side effect. Compared with the vehicle control, the body weights did not change (Fig. 4a). The histopathologic analysis showed that mice treated with SBF-1 exhibited significant reductions in inflammatory cell infiltration, edema, hypersensitivity and vascular congestion in the dermis (Fig. 4f). The positive control CsA also showed a strong therapeutic effect (Fig. 4g).

## Discussion

Excessively activated T lymphocytes have been demonstrated to play a central role in T cell-mediated chronic inflammatory disorders and autoimmune diseases [20]. Hence, they are a potential target for treatment. However, current immunosuppressive agents, such as CsA and dexamethasone, have shown significant safety concerns. Thus, immunosuppressive agents with negligible or acceptable toxicity are needed. In this study, we addressed how SBF-1, which exhibits a strong antitumor activity in various cancer types [14, 21–23], regulated the inflammatory process leading to contact hypersensitivity pathogenesis. Compared with CsA, SBF-1 selectively targeted activated T lymphocytes and showed more potent immunosuppressive activity in vitro and in vivo.

It is believed that the activation and proliferation of T lymphocytes contribute to the inflammatory processes of autoimmune diseases. We found that SBF-1 inhibited Con A-induced cell proliferation in a concentration-dependent manner (Fig. 1b and c). And treatment with 10 nM SBF-1 resulted in a dramatic decrease in the ^3^H-TdR uptake by 100%, showing better efficacy than 1 ⎧M CsA. Similar results were found in anti-CD3 plus anti-CD28 induced T cell proliferation (Fig. 1d and e). In addition, it is notable that SBF-1 did not affect survival of non-activated T cells at the doses mentioned above, indicating that this compound had selectivity for activated T cells to some degree (Fig. 1f and g). When activated, the expressions of CD69 and CD25 on T cells were increased. CD69 is recognized as an early activation marker of stimulated T lymphocytes [24] and CD25 is also a hallmark of activated T cells. In this study, SBF-1 decreased CD25 and CD69 expressions in a concentration-dependent manner (Fig. 2a). Thus, these results suggest that SBF-1 likely inhibits signaling pathways related to T cells activation. Our published results showed that SBF-1 directly inhibited kinase activity of PDK1 and thus down-regulated phosphorylation of AKT [21]. Therefore, we examined the effect of SBF-1 on AKT phosphorylation in Con A-activated T lymphocytes. In fact, we found that SBF-1 markedly reduced phosphorylation of AKT, suggesting that inhibition of AKT signaling events might be associated with impaired T cell response caused by SBF-1 (Fig. 2a). Furthermore, SBF-1 induced apoptosis of activated T cells (Fig. 3a). As typical cytokines of Th1 lineage, IL-2 and IFN-γ have been implicated in the pathogenesis of multiple immune diseases [25]. The mRNA levels of IL-2 and IFN-γ were increased in activated T lymphocytes and SBF-1 dose-dependently reduced their expression. Consistent with this, the secretions of IL-2 and IFN-γ in cell culture supernatant were also inhibited by treatment with SBF-1 (Fig. 3c). Upon activation, T cells proliferate and differentiate into effector T cells and produce cytokines. Overall, the data suggest that the major effect of SBF-1 is on inhibition of T cell activation causing suppression of cytokine production.

Contact hypersensitivity is a T cell-mediated immune response and typically used for the evaluation of immune activity in vivo. We found that SBF-1, when administered by intraperitoneal injection in the induction phase of CHS, significantly suppressed the ear swelling and prevented inflammatory cell infiltration in the edematous sections (Fig. 4). It was important to note that SBF-1 did not cause loss of body weight, perhaps due to its selective effect on activated T lymphocytes.

Taken together, our experiment demonstrated that SBF-1 inhibited the proliferation and activation of T lymphocytes, and induced apoptosis of activated T cells, which is closely associated with its potent down-regulation of AKT signaling pathway. Unlike the classic immunosuppressive agents, SBF-1 showed high activity and low toxicity, owing to its selectivity on activated T lymphocytes, not non-activated T lymphocytes.

## Conclusions

**Fig. 5:**
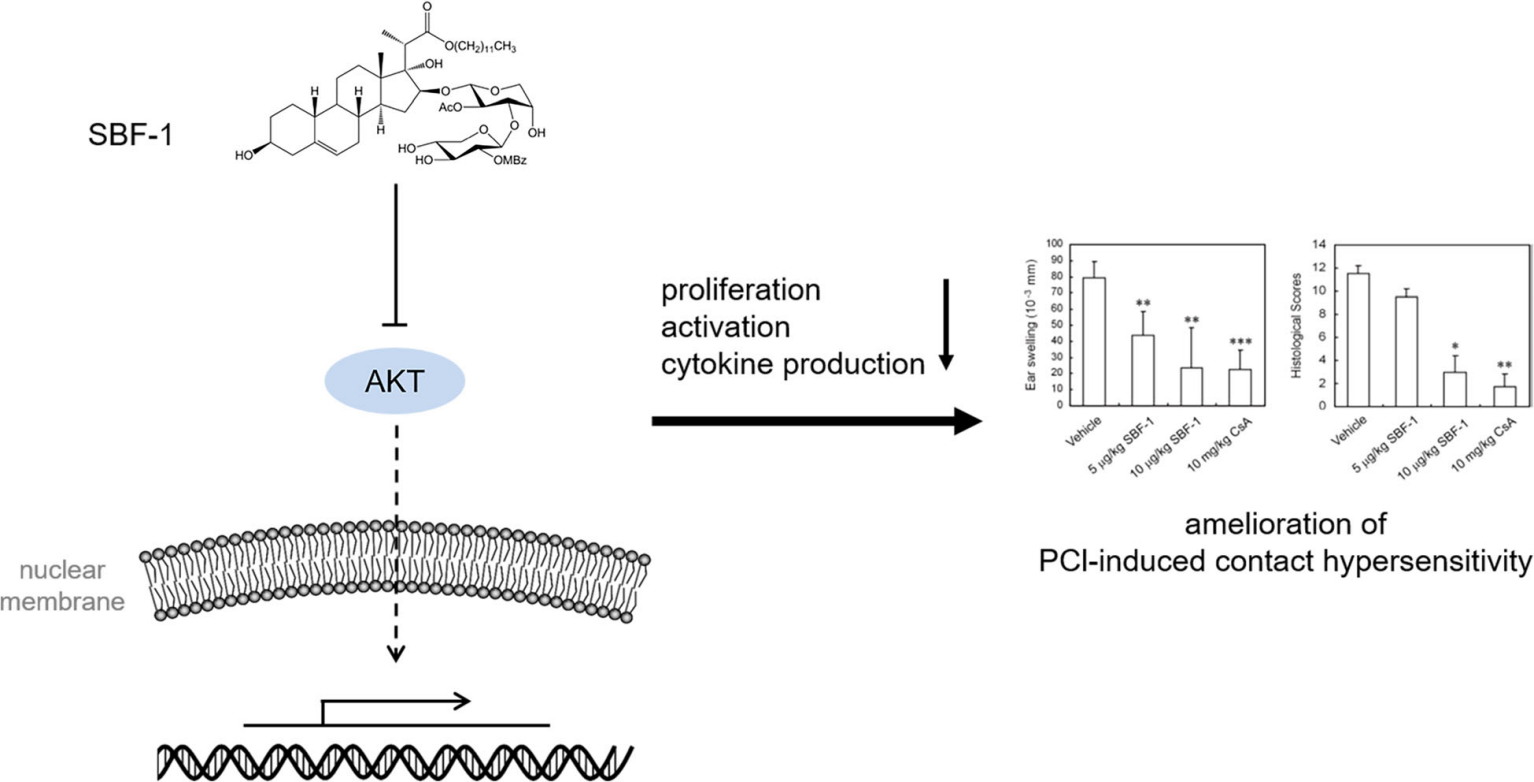
Schematic representation mechanism of SBF-1’s effects on T cells. SBF-1 suppresses T-cell-mediated immune responses in vitro and in vivo by inhibiting T cell proliferation, activation, cytokine production and inducing apoptosis, which is closely related to its blockage of AKT signaling pathways

Our study suggested that SBF-1 suppresses T-cell-mediated immune responses in vitro and in vivo by inhibiting T cell proliferation, activation, cytokine production and inducing apoptosis, which is closely related to its blockage of AKT signaling pathways (Fig. 5).

## Data Availability

The datasets used and/or analyzed during the current study are available from the corresponding author on reasonable request.

## Abbreviations

CHS: Contact hypersensitivity
Con A: concanavalin A
CsA: cyclosporine A
MHC: major histocompatibility complex
MTT: 3-(4,5-Dimethylthiazol-2-yl)-2,5-diphenyltetrazolium bromide
PCl: Picryl chloride
TCR: T cell receptor

## References

[1] Bhattacharyya S, Tobacman JK (2009). Arylsulfatase B regulates colonic epithelial cell migration by effects on MMP9 expression and RhoA activation. Clin Exp Metastasis.

[2] Muro R, Nitta T, Kitajima M, Okada T, Suzuki H (2018). Rasal3-mediated T cell survival is essential for inflammatory responses. Biochem Biophys Res Commun.

[3] Smith-Garvin JE, Koretzky GA, Jordan MS (2009). T cell activation. Annu Rev Immunol.

[4] Speiser DE, Ho PC, Verdeil G (2016). Regulatory circuits of T cell function in cancer. Nat Rev Immunol.

[5] Sell H, Habich C, Eckel J (2012). Adaptive immunity in obesity and insulin resistance. Nat Rev Endocrinol.

[6] Wieland E, Shipkova M (2016). Lymphocyte surface molecules as immune activation biomarkers. Clin Biochem.

[7] Ziegler SF, Ramsdell F, Alderson MR (1994). The activation antigen CD69. Stem Cells.

[8] Flax MH, Caulfield JB (1963). Cellular and vascular components of allergic contact dermatitis. Am J Pathol.

[9] Curzytek K, Kubera M, Szczepanik M, Basta-Kaim A, Leskiewicz M, Budziszewska B, Lason W, Maes M (2013). Crosstalk between contact hypersensitivity reaction and antidepressant drugs. Pharmacol Rep.

[10] Kimber I, Dearman RJ (2002). Allergic contact dermatitis: the cellular effectors. Contact Dermatitis.

[11] Fujimoto Y, Fujita T, Kuramoto N, Kuwamura M, Izawa T, Nishiyama K, Yoshida N, Nakajima H, Takeuchi T, Azuma YT (2018). The role of Interleukin-19 in contact hypersensitivity. Biol Pharm Bull.

[12] Kahan BD (2003). Individuality: the barrier to optimal immunosuppression. Nat Rev Immunol.

[13] Carbone J, del Pozo N, Gallego A, Sarmiento E (2011). Immunological risk factors for infection after immunosuppressive and biologic therapies. Expert Rev Anti-Infect Ther.

[14] Shi B, Wu H, Yu B, Wu J (2004). 23-oxa-analogues of OSW-1: efficient synthesis and extremely potent antitumor activity. Angew Chem Int Ed Engl.

[15] Guggi D, Langoth N, Hoffer MH, Wirth M, Bernkop-Schnurch A (2004). Comparative evaluation of cytotoxicity of a glucosamine-TBA conjugate and a chitosan-TBA conjugate. Int J Pharm.

[16] Li X, Wang X, Jiang H, Zhang G, Tan R, Sun Y, Wu X, Xu Q (2016). Herpetol ameliorates allergic contact dermatitis through regulating T-lymphocytes. Int Immunopharmacol.

[17] Luo Q, Gu Y, Zheng W, Wu X, Gong F, Gu L, Sun Y, Xu Q (2011). Erlotinib inhibits T-cell-mediated immune response via down-regulation of the c-Raf/ERK cascade and Akt signaling pathway. Toxicol Appl Pharmacol.

[18] Cibrian D, Sanchez-Madrid F (2017). CD69: from activation marker to metabolic gatekeeper. Eur J Immunol.

[19] Pompura SL, Dominguez-Villar M. The PI3K/AKT signaling pathway in regulatory T-cell development, stability, and function. J Leukoc Biol. 2018; 10.1002/JLB.2MIR0817-349R 29357116

[20] Perkins DL (1998). T-cell activation in autoimmune and inflammatory diseases. Curr Opin Nephrol Hypertens.

[21] Li W, Song R, Fang X, Wang L, Chen W, Tang P, Yu B, Sun Y, Xu Q (2012). SBF-1, a synthetic steroidal glycoside, inhibits melanoma growth and metastasis through blocking interaction between PDK1 and AKT3. Biochem Pharmacol.

[22] Li W, Ouyang Z, Zhang Q, Wang L, Shen Y, Wu X, Gu Y, Shu Y, Yu B, Sun Y (2014). SBF-1 exerts strong anticervical cancer effect through inducing endoplasmic reticulum stress-associated cell death via targeting sarco/endoplasmic reticulum Ca (2+)-ATPase 2. Cell Death Dis.

[23] Elgehama A, Chen W, Pang J, Mi S, Li J, Guo W, Wang X, Gao J, Yu B, Shen Y (2016). Blockade of the interaction between Bcr-Abl and PTB1B by small molecule SBF-1 to overcome imatinib-resistance of chronic myeloid leukemia cells. Cancer Lett.

[24] Radulovic K, Rossini V, Manta C, Holzmann K, Kestler HA, Niess JH (2013). The early activation marker CD69 regulates the expression of chemokines and CD4 T cell accumulation in intestine. PLoS One.

[25] Schulze-Koops H, Kalden JR (2001). The balance of Th1/Th2 cytokines in rheumatoid arthritis. Best Pract Res Clin Rheumatol.

